# Mobile Phone Intervention Based on an HIV Risk Prediction Tool for HIV Prevention Among Men Who Have Sex With Men in China: Randomized Controlled Trial

**DOI:** 10.2196/19511

**Published:** 2021-04-13

**Authors:** Ke Yun, Zhenxing Chu, Jing Zhang, Wenqing Geng, Yongjun Jiang, Willa Dong, Hong Shang, Junjie Xu

**Affiliations:** 1 NHC Key Laboratory of AIDS Immunology (China Medical University) National Clinical Research Center for Laboratory Medicine The First Affiliated Hospital of China Medical University Shenyang China; 2 Key Laboratory of AIDS Immunology Chinese Academy of Medical Sciences Shenyang China; 3 Key Laboratory of AIDS Immunology of Liaoning Province Shenyang China; 4 Collaborative Innovation Center for Diagnosis and Treatment of Infectious Diseases Hangzhou China; 5 Department of Health Behavior Gillings School of Global Public Health The University of North Carolina at Chapel Hill Chapel Hill, NC United States

**Keywords:** eHealth intervention, high-risk behavior intervention, HIV risk prediction, men who have sex with men

## Abstract

**Background:**

eHealth interventions based on risk stratification have not been extensively applied for HIV behavioral interventions among HIV-negative men who have sex with men (MSM).

**Objective:**

This study aimed to evaluate the efficacy of a mobile phone intervention based on an HIV risk prediction tool in promoting HIV testing and reducing high-risk behavior among HIV-negative MSM in China.

**Methods:**

We performed a mobile phone–based randomized controlled clinical trial for 12 weeks. A comprehensive intervention package deployed on Jinshuju—an online survey platform—was developed and consisted of 4 components: (1) a validated HIV risk prediction tool that provides information on personalized risk reduction interventions; (2) a map of individualized HIV testing facilities based on their geographic location; (3) a QR code for free resources on HIV prevention, including condoms and HIV self-testing kits; and (4) general resources for HIV health education. MSM participants recruited from WeChat/QQ groups were randomly assigned to the intervention or control group at a 1:1 ratio. The staff sent the QR code for the comprehensive intervention package to MSM in the intervention group over WeChat and sent the QR code only for the resources on HIV health education to those in the control group. At baseline and 12-week follow-up, data on HIV-related risk behavior and HIV testing behavior were collected through the Jinshuju online survey platform.

**Results:**

In total, 192 MSM were recruited and assigned to the intervention or control group (n=96 each). At week 12, the total clinical trial retention rate was 87.5%. The number of male sexual partners of the MSM in the past 3 months was significantly lower in the intervention group than in the control group (3.51, SD 4.1 vs 6.01, SD 11.4, respectively; mean difference −2.5; 95% CI −5.12 to 0.12; *P*=.05); the rate of condom use with casual sexual partners was higher in the intervention group than in the control group (87%, n=66/76 vs 70%, n=54/77 respectively; odds ratio 2.81, 95% CI 1.23-6.39; *P*=.01). The proportion of individuals intending to undergo HIV testing after in the following 30 days was marginally higher in the intervention group than in the control group (90%, n=77/86 vs 79%, n=65/82 respectively; odds ratio 2.20, 95% CI 0.90-5.35; *P*=.07). The incremental cost-effectiveness ratio of eHealth intervention was US $131.60 on reducing 1 sexual partner and US $19.70 for a 1% increment in condom usage with casual partners.

**Conclusions:**

A comprehensive intervention based on an HIV risk prediction tool can reduce the number of male sexual partners among MSM and increase the rate of condom use with casual partners. Hence, this intervention is a very promising preventive strategy for HIV among MSM, especially in areas with a prominent HIV epidemic.

**Trial Registration:**

Chinese Clinical Trial Registry ChiCTR1800017268; http://www.chictr.org.cn/showprojen.aspx?proj=29271

## Introduction

Men who have sex with men (MSM) are a significant target population for the prevention and control of HIV infections worldwide [1]. In China, although traditional sexual behavioral interventions, such as promoting abstinence and condom use, have played an essential role in curbing the HIV epidemic, HIV acquisition among MSM, especially young MSM, remains poorly controlled [2]. In 2015, HIV prevalence among MSM was 8%, and its incidence was 5.61 cases per 100 person-years [3]. Young MSM aged 20-29 years may be disproportionately affected because they accounted for 47.0% of the 120,371 HIV cases recorded during 2006-2015 in China [4].

In recent years, internet-based dating websites or smartphone apps have been increasingly used by MSM to seek sexual partners [5]. In the United States, a study reported that among 195 MSM aged 19-24 years, 75% of participants reported having sexual encounters with individuals through these apps [6]. Similarly, in China, out of 353 MSM, almost 70% reported having sexual experiences with men they met on the internet [7]. Furthermore, among 375 young MSM, 70% reported having unprotected anal intercourse with partners from the internet-based dating app Grindr and perceived a low risk of HIV acquisition [8]. Fortunately, internet-based eHealth interventions have emerged as prominent tools for preventing HIV acquisition among MSM owing to their convenience and low cost [9]. Therefore, considering the popularity of internet-based platforms for interacting with sexual partners, social media may be leveraged to deliver behavioral interventions, especially among young MSM, who are not only particularly vulnerable to an HIV infection compared to those in other age groups [10] but also willing to receive HIV prevention interventions via smartphone apps [11].

We previously developed and validated an HIV risk prediction model and constructed a social media platform–based HIV risk assessment tool [12]. Based on the HIV risk assessment tool, we then developed a comprehensive intervention package for MSM to estimate their probability of acquiring an HIV infection and to provide personalized and tailored information on HIV testing and reducing behavioral risks. This study aimed to assess the preliminary efficacy of an eHealth intervention, based on a previously developed and validated HIV risk prediction tool [12], in reducing the HIV risk and promoting HIV testing among MSM in China.

## Methods

### Study Population and Design

**Figure 1:**
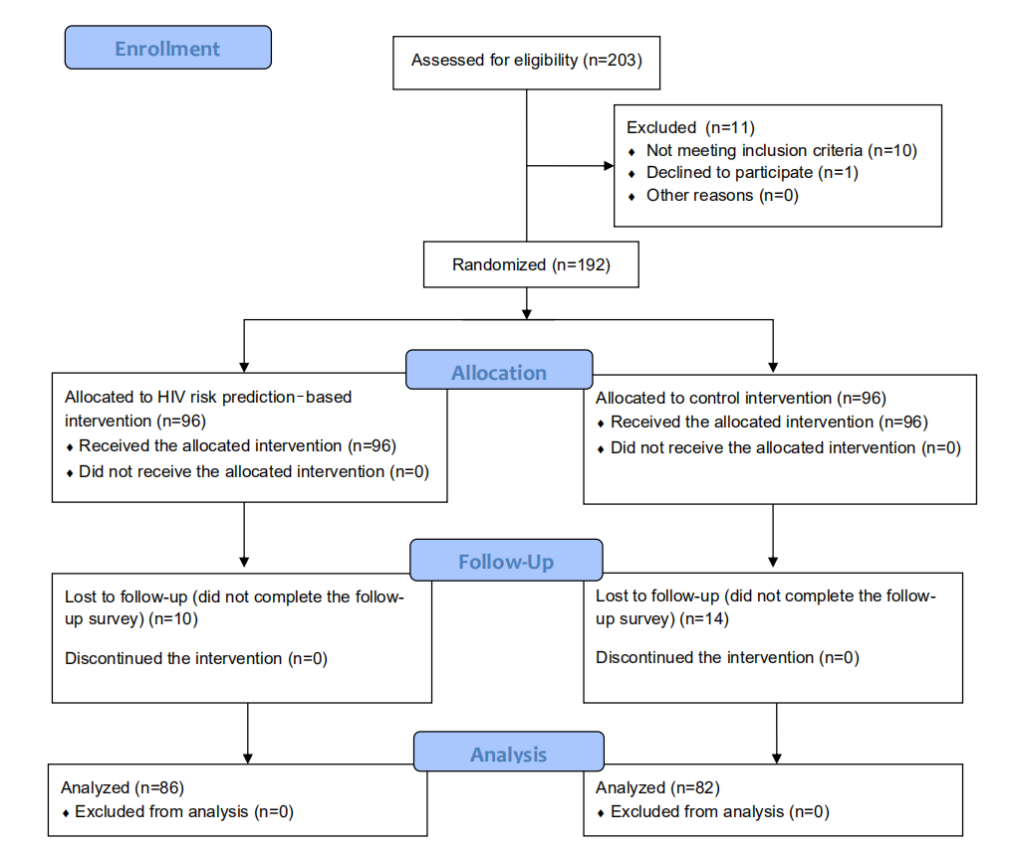
Consolidated Standards of Reporting Trials flowchart for the recruitment of participants who are men who have sex with men in Shenyang, China.

This 2-arm, parallel, randomized, double-blinded clinical trial was conducted in accordance with the Consolidated Standard of Reporting Trials guidelines [13] for randomized controlled trials (Multimedia Appendix 1) and the Checklist for Reporting Results of Internet E-Surveys [14] for online surveys (Multimedia Appendix 2). Figure 1 shows a Consolidated Standard of Reporting Trials flow diagram of the study design. From October 2017 to March 2018, participants were recruited through an advertisement on a popular WeChat/QQ group for MSM in China with the assistance of Shenyang Sunshine Working Group, a local MSM community-based organization (CBO). Following the completion of a web-based screening questionnaire, the computer algorithm immediately provided an eligibility assessment. The inclusion criteria were as follows: men who had anal or oral intercourse with men in the previous year, had not been diagnosed with an HIV infection, owned an Android or Apple smartphone and a WeChat account, were aged ≥18 years, provided written informed consent, and were able to comprehend written Chinese (Mandarin). The exclusion criteria were as follows: not having a WeChat account and having tested positive for HIV. Informed consent was received from all MSM involved in the survey.

### Randomization and Study Procedures

All eligible MSM were randomly assigned to an intervention group and a control group on Microsoft Excel 2010 at a 1:1 ratio after obtaining informed consent. Participants and investigators were both blinded to group allocation. At baseline and prospective follow-up, MSM included in our study received a push notification with a QR code for a link to the questionnaire through the study’s WeChat account. These 28-item questionnaires were displayed on a webpage pertaining to high-risk sexual behavior and HIV testing in the previous 3 months and contained questions on the number of sexual partners, condom usage, practices related to unprotected anal intercourse, and intentions to undergo HIV testing in the following 30 days. Before administering the questionnaire in an open-field survey, a small-scale pilot study was conducted to ensure the reliability and validity of the survey instrument. Participants were required to answer all questions, and we programmed skip patterns, which are a feature of the survey platform, to reduce participant burden. The respondents could review and change their answers before submission. Participants who completed the web-based questionnaire survey received a ¥20 (approximately US $3) subsidy to thank them for their participation. Implementation was carried out as follows. First, after the completion of the recruitment screening questionnaire, participants were asked to add the study’s WeChat account as an active friend and then send screenshots of the completed questionnaire to the study WeChat account. After the staff assessed the integrity of the questionnaire, they transferred the subsidy to the participants through WeChat transfer. The staff used the WeChat nickname and WeChat ID to ensure that the questionnaire responders and recipients are the same individuals. Every time the participants completed the questionnaire, they received the subsidy through the app.

### Intervention

The theoretical framework for the comprehensive intervention package used in our study is the AIDS Risk Reduction Model established in 1990 [15]. The staff sent the link to the QR code for the comprehensive intervention package to MSM in the intervention group over WeChat, and a link to a QR code for a webpage with resources on HIV/AIDS health education was sent to MSM in the control group. The intervention package included 4 components that were organized by the intervention index interface deployed on the Jinshuju data management platform, which included the following: (1) an HIV risk prediction tool used for personalized evaluation of the HIV risk [12], feedback on risk behavior, and tailored suggestions for risk reduction; (2) recommendations for HIV testing facilities based on the user’s location, which were churned by the questionnaire system backend after participants authorized the app to access their geographic location data; (3) web-based forms to receive order-free prevention resources on demand via mail, including condoms and HIV self-testing kits; and (4) an HIV health education website that contains information on HIV and associated hazards, the situation of the epidemic among MSM in China, and self-protection against HIV. All the information related to the intervention was presented on an external website. Detailed screenshots of the intervention interface are presented in Multimedia Appendices 3-7. Every 4 weeks, information related to the intervention was sent again as a reminder to participants in both groups. Throughout the study period, MSM used WeChat as they did before the study.

To ensure the accuracy of the questionnaire survey, 3 measures were taken to avoid duplicate recruitment of the same individual in the study. First, participants could only complete the deployed questionnaire survey through WeChat, and secondary sharing of questionnaire links was prohibited. The questionnaire and data management system we used in this study restricted access to the specific survey pages in accordance with the IP address. Second, although WeChat nicknames are variable and dynamic, each WeChat account has a unique ID. On visiting the questionnaire, the survey system asked participants to authorize access to their personal WeChat ID information, and the unique WeChat ID was stored together with the survey results. Consequently, we could identify and verify the uniqueness of individuals by their WeChat ID. We did not use cookies to assign a unique user identifier to each client computer. Third, in the data processing stage, we deleted duplicate records from the same ID and records with identical responses, such as personal information and reported high-risk behavior. The first entry was retained for analysis.

### Primary Outcomes and Related Definitions

The primary outcomes of this study were the proportion of HIV testing, condom usage, anal intercourse in the past 3 months, and intention to undergo HIV testing in the next 30 days. The secondary outcome was the number of male sexual partners in the past 3 months. These outcomes were measured with a self-reported web-based questionnaire at 12 weeks post baseline. We defined an interval 7 days before and after the follow-up time point as the effective survey window for the follow-up time point. The observation period was determined in accordance with the survey experience of sexual behavioral among the MSM included in our study and previous randomized controlled trials [9]. Study subjects who failed to participate in the follow-up questionnaire during the survey window were considered as being lost to follow-up. The rate of being lost to follow-up was defined as the ratio of subjects who failed to participate in the 12-week follow-up to the total number of MSM recruited at baseline. Adherence was assessed by the percentage of individuals who visited the intervention index webpage (based on their IP addresses) out of the total number of participants in the intervention and control groups who completed the baseline questionnaire.

### Sample Size Calculation

We assumed that the intervention would be more effective than the resources on HIV/AIDS health education provided to the control group. According to our pilot survey, the assumption parameters were as follows: the average number of sexual partners in the past 3 months (primary study outcome) was 7 in the control group and 5 in the intervention group, and the SD for sample size estimation was 5. Other specific parameters are listed as follows: the degree of certainty (1-β) was 80%, the significance level (Cronbach *α*) was .05, and 20% of subjects were lost to follow-up. Therefore, we intended to recruit 200 subjects. The sample size was estimated using the independent samples *t* test module of PASS 2008 (NCSS LLC).

### Cost Measurement and Cost-Effectiveness Criteria

The cost was estimated from the social perspective, and only the direct cost associated with the intervention was considered. The cost, including human capital cost, CBO recruitment and referral fees, communication fees, electricity fees, cost of condoms and lubricants, cost of HIV self-testing strips, postage fees, cost of the online questionnaire system, compensation for the participants, and stationary, was estimated in accordance with the clinical trial. The cost-effectiveness ratio (CER) and incremental cost-effectiveness ratio (ICER) were calculated as the economic evaluation indicators. The World Health Organization cost-effectiveness criteria were used for economic analysis, which state that interventions with an ICER of <0 are effective and cost-effective, those with an ICER less than the average per capita GDP for a particular country or region are considered highly cost-effective, those with an ICER of <3-fold average per capita GDP are still considered cost-effective, and those that exceed this level are considered non–cost-effective [16]. The average per capita GDP for China in 2017 was US $8497, which was considered the threshold indicating the cost-effectiveness of the intervention in this study.

### Statistical Analysis

Data were collected using the Jinshuju online survey platform (https://jinshuju.net).

Only completed eligible questionnaires were analyzed. Participants were required to answer all questions to minimize missing data and the need for imputation. Participants lost to follow-up were not included in the final intervention effect analysis. The chi-square test or Fisher exact probability test was used to compare categorical variables, and the independent samples *t* test was used to compare continuous data. All statistical analyses were performed using SAS (version 9.4, SAS Institute). Findings were considered significant when *P*<.05

### Ethics Approval and Informed Consent

The Ethics Review Committee of the First Affiliated Hospital of China Medical University (Shenyang, China) approved the study protocol, investigation procedures, and questionnaires (approval# 2018-175-2). Written informed consent was obtained from all subjects before their participation in the questionnaire survey. WeChat IDs and self-reported dates of birth were used for individual identification to ensure that the participants’ privacy was effectively protected.

## Results

### Recruitment, Screening, and Prospective Follow-up of Subjects

Between October 2017 and March 2018, of the 587 MSM who clicked on the recruitment advertisement, 203 completed the final screening questionnaire, and 192 were eligible for randomization (Figure 1). The mean age of the study subjects included was 28.5 (SD 7.9) years in the intervention group and 26.9 (SD 8.1) years in the control group. Among them, 113 (58.9%) had a Shenyang household registration, 114 (59.4%) had a monthly income of >¥3000 (approximately US $437), 95 (49.5%) were single; 113 (58.9%) had a high school or higher degree, 74 (39.4%) were employed, 112 (58.3%) looked for sexual partners on the internet or on social media, and 173 (90.1%) had their first sexual experience at an age of <30 years. In the past 3 months, 176 (91.7%) participants exhibited same-sex sexual behavior, 120 (62.5%) had at least 2 male sexual partners, 103 (53.6%) had practiced unprotected anal intercourse, 77 (40.1%) had unprotected receptive anal intercourse, and 82 (42.7%) engaged in group sex. We observed no significant difference in the aforementioned baseline characteristics between the intervention group and control groups (Table 1).

**Table 1:** Baseline sociodemographic and behavioral characteristics of men who have sex with men in Shenyang, China, in the clinical trial (N=192).

Variable		Intervention group (n=96)	Control group (n=96)	*P* value
Age (years), mean (SD)		28.5 (7.9)	26.9 (8.1)	.18
**Local residents, n (%)**				.30
	Yes	60 (63)	53 (55)	
	No	36 (38)	43 (45)	
**Monthly income (** **¥** **), n (%)**				.14
	≤3000	34 (35)	44 (46)	
	>3000	62 (65)	52 (54)	
**Marital status, n (%)**				.67
	Single	49 (51)	46 (48)	
	Married or cohabiting with a partner	47 (49)	50 (52)	
**Education level, n (%)**				.95
	Below high school	40 (42)	37 (39)	
	High school	51 (53)	52 (55)	
	College and above	5 (5)	5 (5)	
**Occupation, n (%)**				.64
	Worker/staff	38 (40)	36 (39)	
	Business	35 (37)	27 (29)	
	Student	15 (16)	19 (21)	
** **	Other	8 (8)	10 (11)	
**Age at first sexual experience (years), n (%)**				.22
	<30	84 (88)	89 (93)	
	≥30	12 (13)	7 (7)	
**Main venue to seek sexual partners, n (%)**				.42
	Smartphone apps/web-based dating platforms	51 (53)	61 (64)	
	Park/public bath/public toilet	6 (6)	4 (4)	
	Bar/club	3 (3)	1 (1)	
	Other	36 (38)	30 (31)	
**Displaying same-sex sexual behavior in the past 3 months, n (%)**				.30
	Yes	90 (94)	86 (90)	
	No	6 (6)	10 (10)	
**Having ≥2 male sexual partners in the past 3 months, n (%)**				.77
	Yes	61 (64)	59 (62)	
	No	35 (37)	37 (39)	
**Having unprotected anal intercourse in the past 3 months, n (%)**				.11
	Yes	57 (59)	46 (48)	
	No	39 (41)	50 (52)	
**Having unprotected receptive anal intercourse in the past 3 months, n (%)**				.66
	Yes	40 (42)	37 (39)	
	No	56 (58)	59 (62)	
**Engaging in group sex in the past 3 months, n (%)**				.70
	Yes	45 (47)	37 (39)	
	No	51 (53)	59 (62)	
Number of male sexual partners in the past 3 months, mean (SD)		3.3 (3.4)	3.9 (4.7)	.33

### Adherence to the Intervention

In the past 3 months, the rate of page visits in the intervention group remained stable (68%, 52%, and 65% in months 1, 2, and 3, respectively), whereas those in the control group displayed an apparent downward trend (60%, 39%, and 18% in months 1, 2, and 3, respectively). For the 3 intervention modules in the intervention group, the risk assessment module had the highest page click rate.

### Efficacy of the Intervention

At week 12 of prospective follow-up, 168 questionnaires were collected (86 and 82 in the intervention and control groups, respectively), and the clinical trial retention rates were 90% (n=86/96) and 85% (n=82/96) for the intervention and control groups, respectively.

We observed no significant difference in the proportion of MSM who underwent HIV testing in the past 3 months. The proportion of MSM who intended to undergo HIV testing in the following 30 days was slightly higher in the intervention group than in the control group (90%, n=77/86 vs 79%, n=65/82, respectively; odds ratio [OR] 2.20, 95% CI 0.90-5.35; *P*=.07). Condom usage among casual sexual partners in the past 3 months was significantly higher in the intervention group than in the control group (87%, n=66/76 vs 70%, n=54/77, respectively; OR 2.81, 95% CI 1.23-6.39; *P*=.01). We observed no significant difference in the proportion of participants engaging in passive anal intercourse with or without a condom in the past 3 months and those engaging group sex. The number of male sexual partners in the past 3 months was significantly lower in the intervention group than in the control group (3.51, SD 4.1 vs 6.01, SD 11.4, respectively; mean difference=−2.5, 95% CI −5.12 to 0.12; *P*=.05) (Table 2).

**Table 2:** Effect of the eHealth intervention based on the HIV risk prediction tool for men who have sex with men on HIV-related high-risk behaviors and intentions to undergo HIV testing (N=168).

HIV-related behavior	Intervention group (n=86)	Control group (n=82)	Effect size, OR^a^ or mean difference^b^ (95% CI)	*P* value
Proportion of participants having undergone HIV testing in the past 3 months, n (%)	75 (87)	68 (83)	1.30^a^ (0.55 to 3.09)	.55
Proportion of participants who intend to undergo HIV testing in the following 30 days, n (%)	77 (90)	65 (79)	2.20^a^ (0.90 to 5.35)	.07
Proportion of participants who used condoms in the past 3 months with causal sexual partners, n (%)	66 (87)	54 (70)	2.81^a^ (1.23 to 6.39)	.01
Proportion of participants who had passive anal intercourse in the past 3 months, n (%)	52 (61)	59 (72)	0.57^a^ (0.30 to 1.10)	.09
Proportion of participants who had unprotected passive anal intercourse in the past 3 months, n (%)	23 (27)	29 (35)	0.65^a^ (0.34 to 1.26)	.21
Proportion of participants engaging in group sex in the past 3 months, n (%)	6 (7)	7 (9)	0.80^a^ (0.26 to 2.50)	.14
Number of male sexual partners in the past 3 months, mean (SD)	3.5 (4.1)	6.0 (11.4)	–2.50^b^ (–5.12 to –0.12)	.05

^a^OR: odds ratio and 95% CI values have been used to indicate the effect size.

^b^Mean difference and 95% CI values have been used to indicate the effect size.

### Cost-Effectiveness Analysis

The total cost for the intervention group was US $2577. The CER for the reduction in male sexual partners was US $734.20, and the ICER was US $131.60. The CER for the promotion of condom usage with casual partners was US $29.70, and the ICER was US $19.70. Both ICERs were lower than the cost-effectiveness threshold for China (Table 3).

**Table 3:** Cost-effectiveness analysis of an eHealth intervention based on an HIV risk prediction model for men who have sex with men in China.

Groups		Effect	Cost (US $)	Cost/effect (US $)	Incremental cost-effectiveness ratio (US $)
**Reduction in the number of sexual partners**					
	Control, n	6.01	2248	374.0	N/A^a^
	Intervention, n	3.51	2577	734.2	131.6
**Promotion of condom usage with casual sexual partners**					
	Control, %	70	2248	32.1	N/A
	Intervention, %	87	2577	29.7	19.7

^a^N/A: not applicable.

## Discussion

### Principal Findings

To our knowledge, this study is the first to assess the efficacy of an eHealth intervention based on an HIV risk prediction tool for the reduction of risk behavior and promotion of HIV testing among MSM and is a necessary step prior to the implementation of the predictive model in clinical practice. The number of male sexual partners in the intervention group significantly decreased during the study period, while the rate of insistence on condom usage among casual male sexual partners significantly increased. These findings indicate that eHealth interventions based on risk prediction might promote healthy sexual behavior among MSM.

This study found that a comprehensive online intervention based on risk assessment can significantly reduce the number of sexual partners among MSM. Previous studies have indicated that MSM with multiple sexual partners displayed continuous inconsistencies in terms of knowledge and behavior [17], probably owing to the insufficiency of conventional educational approaches to modify high-risk behaviors. In our study, the effectiveness of interventions in reducing the number of sexual partners may be attributed to an enhancement in risk perception through the personalized web-based risk evaluation component [18]. Therefore, the large-scale promotion and application of a personalized HIV risk prediction tool on social media platforms may increase risk perception and would be expected to reduce sexual network density among MSM.

Furthermore, we found that the risk prediction–based eHealth intervention could significantly increase condom usage with casual sexual partners among MSM. Pan et al [19] reported that MSM who seek sexual partners through web-based or social media platforms usually had more sexual partners, and notably more casual sexual partners, than those seeking sexual partners at other venues. Having unprotected intercourse with casual partners is an independent risk factor for HIV acquisition among MSM [19]. Thus, this intervention strategy aimed at improving HIV risk perception among MSM, and targeted interventions are useful for reducing the HIV risk among MSM who seek sexual partners on web-based or social media platforms [20]. In addition, the promotion of HIV testing is vital for preventing HIV infections, considering the low HIV testing rates in China. However, we observed only a marginal increase in the rate of participants intending to undergo HIV testing in the following 30 days in our study. One study performed in Peru [21] reported that peer-mentored social media community–based interventions may improve the HIV testing rates among MSM. Therefore, the combination of social media community–based interventions with our risk prediction tool may increase the levels of HIV testing and reduce high-risk behavior in the future.

The clinical trial retention rate in our study approached 87.5%, which was comparable to a cluster randomized controlled trial on web-based peer education with a social media platform–based intervention to increase the HIV testing rate among MSM in Peru (90% retention rate at 12-week follow-up) [21]. This finding revealed that internet-based recruitment and the implementation of an eHealth intervention based on an HIV risk prediction tool are feasible. The higher clinical trial retention rates might be explained by the following reasons. First, monetary incentives in our study could improve the response enthusiasm to online surveys. Second, CBOs are familiar with MSM community members, understand their needs, and provide a trusted environment for communication among MSM. Generally, monetary incentives and CBOs could promote online recruitment and retention of MSM in internet-based studies aimed at improving service efficiency and effectiveness in preventing HIV infections [22,23]. Moreover, our results suggest that eHealth interventions based on risk prediction may help increase condom usage and decrease the number of male sexual partners among MSM. Compared with traditional facility-based HIV testing, our intervention could provide timely alerts to MSM with high-risk behaviors to make risk-reduction decisions and to provide information and resources on HIV prevention in a personalized manner [24]. Furthermore, the comprehensive online intervention model based on risk assessment used in this study was associated with a low cost. The cost-effectiveness of this intervention may be attributed to the conductance of this study on a social media platform, thus saving on housing costs and costs associated with other facilities. Additionally, an eHealth intervention can be extended to MSM who may not have time, resources, or the motivation to seek in-person preventive services.

### Limitations

This study has several limitations, which should be considered when extrapolating the results of our study. First, information collected from the questionnaires was self-reported, and laboratory data on sexually transmitted diseases (eg, HIV and syphilis) were not collected. Thus, we could not evaluate whether the intervention strategy had an influence on HIV or sexually transmitted infections among MSM. Second, some MSM may not be frequent internet users, such as those with a low educational background and older MSM; therefore, they may not be well-suited for eHealth interventions. Therefore, other offline interventions should be developed as essential alternatives to web-based interventions to be extended to these groups. Third, the 12-week study duration may have contributed to a recall bias and telescoping errors for data collected through the web-based survey. Moreover, the extent to which MSM, who are familiar with local CBOs, who agree to participate in preventive interventions is different from MSM who are not familiar with local CBOs, thus resulting in a potential selection bias.

### Generalizability of the Findings

This study is applicable to MSM who use mobile phones and are willing to accept mobile phone–based interventions. The inclusion criterion of having a WeChat account limited the representativeness of the study population among the nationwide MSM population in China because MSM with a low educational background and older adult MSM may not be well-suited for mobile phone–based interventions. Therefore, our findings may not be generalizable to MSM not owning smartphones or to those who are not receptive to mobile phone–based interventions.

### Implications for Practice

These findings further the current understanding of the efficacy of comprehensive interventions based on HIV risk prediction models—which are delivered through social media platforms—on HIV-related behavioral changes among MSM, and provides a new paradigm for health interventions for MSM and more opportunities for HIV surveillance and treatment, which have considerable implications and prospects.

### Future Prospects

Although our study demonstrates the efficacy of HIV risk prediction–based mobile phone interventions in promoting HIV testing and reducing high-risk behavior among MSM in China, the efficacy of this intervention in reducing the incidence of HIV or other sexually transmitted infections remains unclear owing to the lack of corresponding laboratory data and biological endpoints. Thus, future studies should collect laboratory data on HIV or other sexually transmitted infections and assess the efficacy of the intervention on epidemics of HIV or sexually transmitted infections among MSM. Furthermore, data on the proportion of MSM who are sex workers or MSM who have had female sexual partners should be collected in a future study to further the current understanding of the interaction of sexual networks among MSM and to verify the reliability of the survey data. Eventually, considering the important role of monetary incentives and CBOs in MSM recruitment and clinical trial retention, monetary incentives and mechanisms facilitating or supporting CBOs’ engagement in effective and sustainable HIV/AIDS prevention programs should be considered in future peer studies.

### Conclusions

A mobile phone–based intervention based on an HIV risk prediction tool is feasible for MSM in China; this intervention could reduce the number of sexual partners and promote condom usage with casual sexual partners among MSM, thus providing a novel, convenient, and accessible intervention paradigm for this key population.

## Appendix

Multimedia Appendix 1 CONSORT-EHEALTH checklist (V 1.6.1).

Multimedia Appendix 2 Checklist for Reporting Results of Internet E-Surveys.

Multimedia Appendix 3 Intervention index interface.

Multimedia Appendix 4 MSM risk assessment and tailored suggestions.

Multimedia Appendix 5 Application of free HIV self-testing kits and condoms.

Multimedia Appendix 6 Surrounding medical facility recommendation by geographical location.

Multimedia Appendix 7 HIV_AIDS health education.

## Abbreviations

CBO: community-based-organization
CER: cost-effectiveness ratio
ICER: incremental cost-effectiveness ratio
MSM: men who have sex with men
OR: odds ratio
