# Supplementary figures and images for: Mobile Phone Intervention Based on an HIV Risk Prediction Tool for HIV Prevention Among Men Who Have Sex With Men in China: Randomized Controlled Trial
Source: JMIR Mhealth Uhealth. 2021 Apr 13;9(4):e19511. doi: 10.2196/19511 (PMC8080142; doi:10.2196/19511)

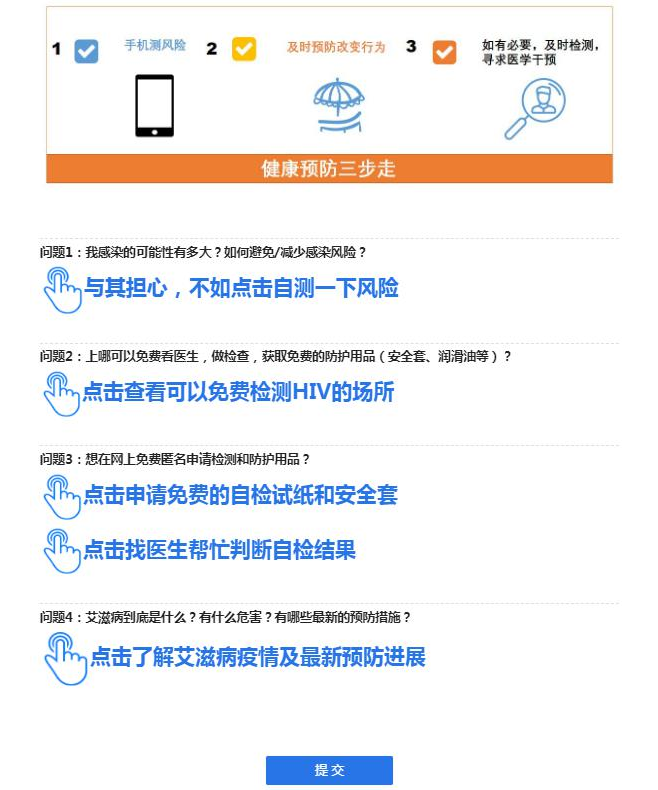

Supplement: Multimedia Appendix 3 [file mhealth_v9i4e19511_app3.png]

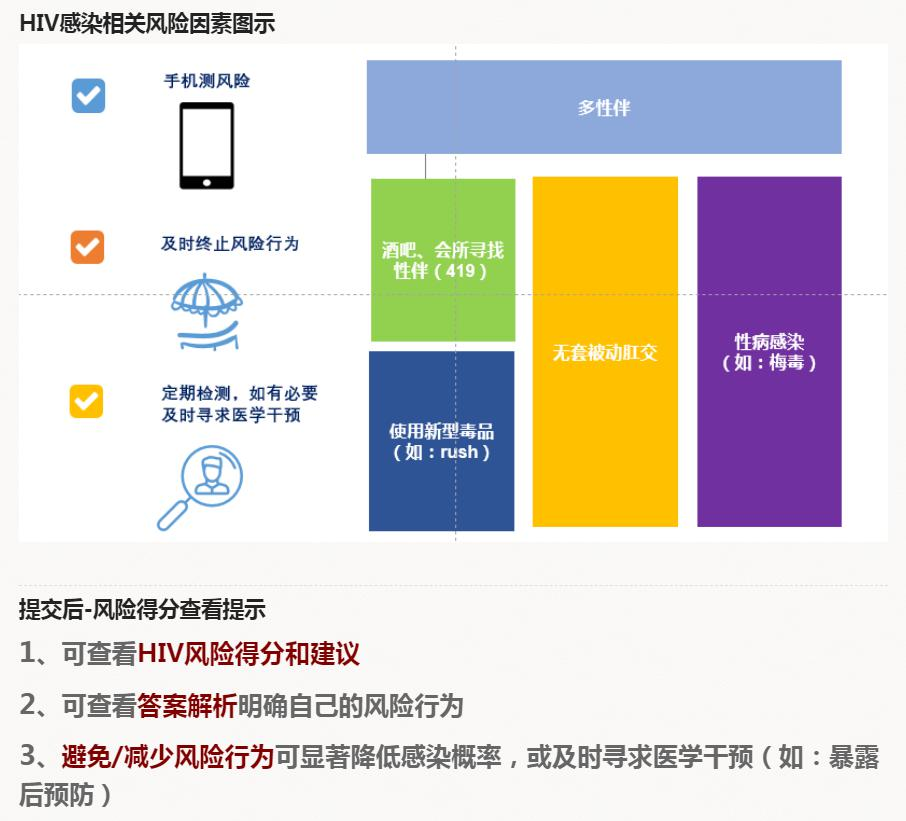

Supplement: Multimedia Appendix 4 [file mhealth_v9i4e19511_app4.png]

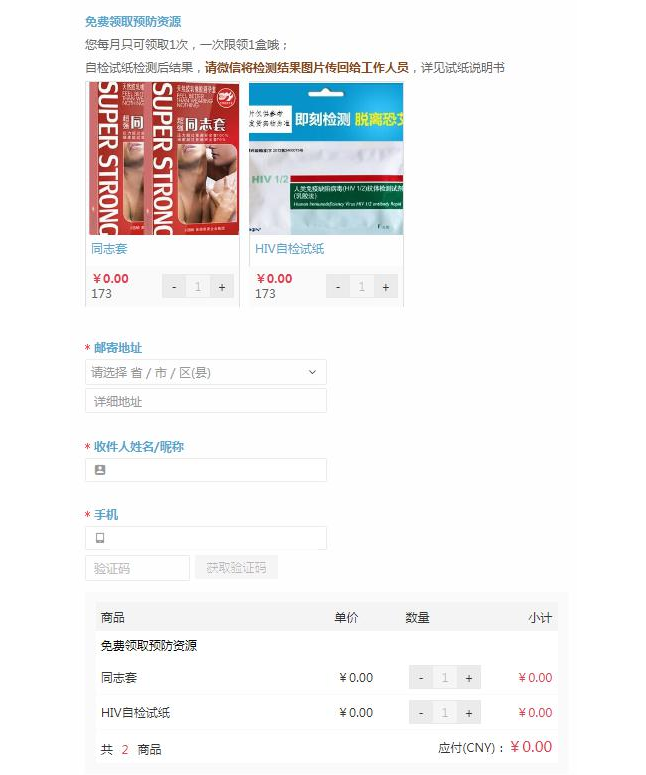

Supplement: Multimedia Appendix 5 [file mhealth_v9i4e19511_app5.png]

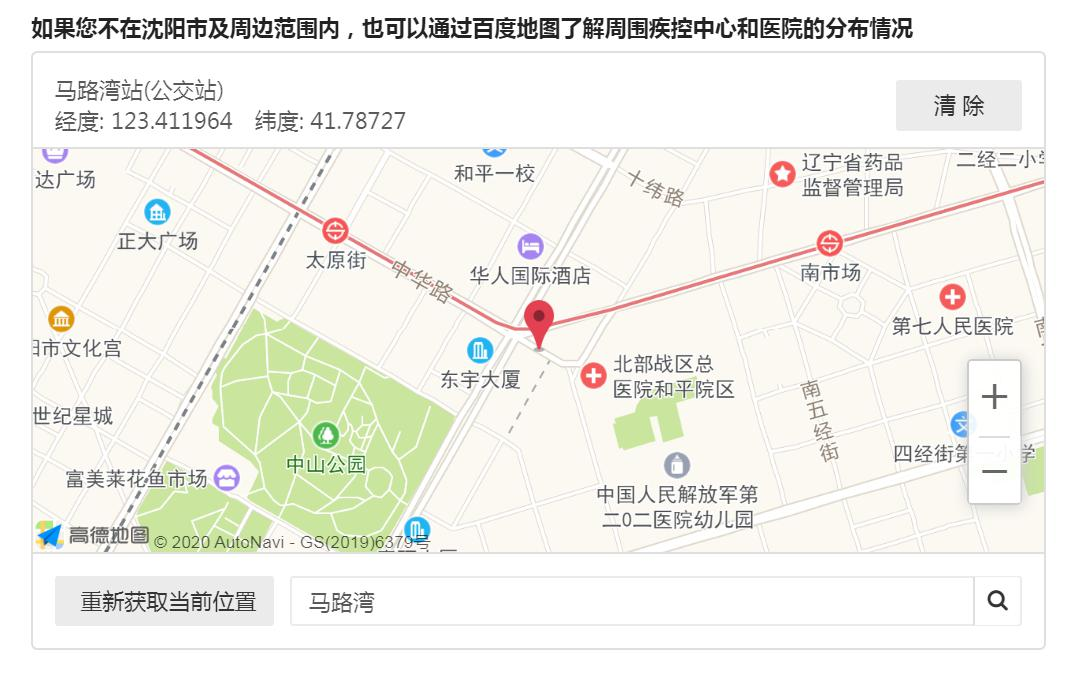

Supplement: Multimedia Appendix 6 [file mhealth_v9i4e19511_app6.png]

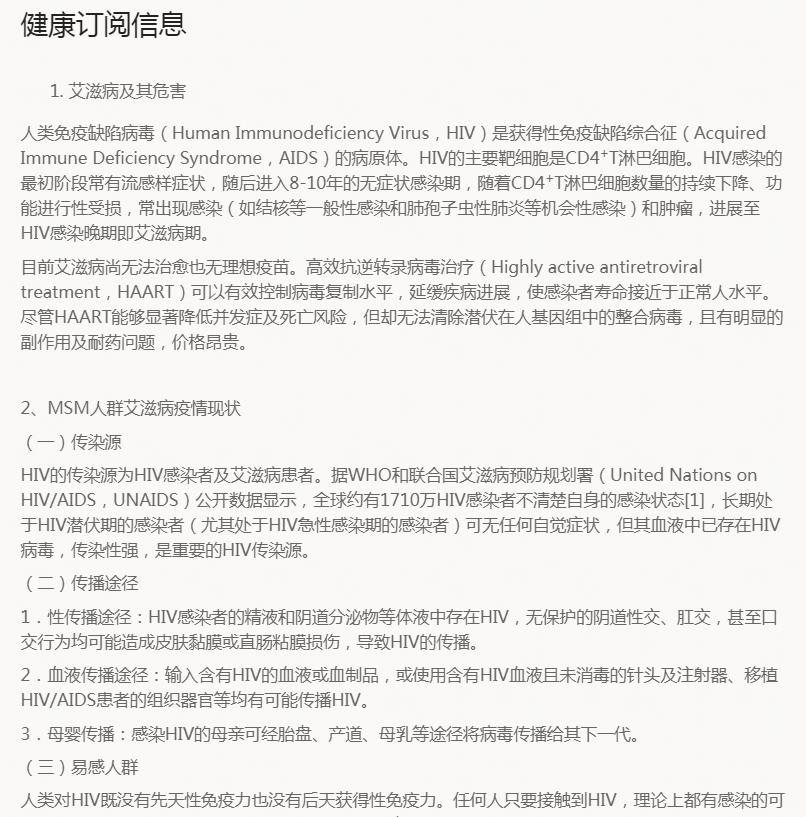

Supplement: Multimedia Appendix 7 [file mhealth_v9i4e19511_app7.png]
